# Partial coherence of inelastically scattered electrons in transmission electron microscopy

**DOI:** 10.1093/jmicro/dfag020

**Published:** 2026-03-25

**Authors:** Budhika Gehan Mendis

**Affiliations:** Department of Physics, Durham University, South Road, Durham DH1 3LE, UK

**Keywords:** inelastic electron scattering, partial coherence, coherence volume, dynamical electron diffraction, time-dependent perturbation theory, energy-filtered diffraction

## Abstract

Coherence of electron waves is central to transmission electron microscopy. However, the mechanism underlying partial loss of coherence during inelastic scattering has been a perplexing problem for many decades. Here it is shown that the inelastic collision time is the key parameter governing coherence. Large energy transfers have short collision times and an inversely decaying coherence length. It is also shown that the inelastic coherence volume is highly elongated along the electron beam direction. Plasmon excitation and inter-band transition events, therefore, have only a minor effect on dynamical diffraction, a fact confirmed by energy-filtered measurements on [001]-oriented SrTiO_3_.

## Teaser

The collision time is proposed as governing coherence in inelastic scattering. Large energy transfers have short collision times and an inversely decaying coherence length. It is also shown that the inelastic coherence volume is highly elongated along the electron beam direction. Electron diffraction data on SrTiO3 confirm these conclusions.

## Introduction

In transmission electron microscopy (TEM), electron beam coherence manifests in a variety of ways. For example, limitations imposed by the spatial and temporal coherence of the electron beam source on high-resolution electron microscopy lattice imaging are well documented [1]. On the other hand, in high-angle annular dark field imaging, coherence is deliberately destroyed by integrating the signal over an annular detector [2,3]. Coherent Bragg scattering within a crystal is also exploited in channelling enhanced X-ray microanalysis [4] as well as magnetic circular dichroism measurements [5], although the so-called ‘preservation of elastic contrast’ is often an undesirable feature in composition analysis along zone-axis orientations [6] and Compton scattering [7]. Less well understood is the coherence of electrons that have lost some amount of energy to the sample. Early pioneering studies on diffraction contrast of small-angle inelastic scattered electrons [8] and interference fringes in plasmon energy loss-filtered electron holography [9–11] showed that a certain degree of coherence was preserved. Measurements on phase contrast [12] and Fresnel fringing at the specimen edge [13] have shown the coherence length to decrease monotonically with energy loss. Egerton [14] has proposed that when the characteristic scattering angle for the inelastic event exceeds the Bragg angle, the electrons are fully incoherent and no longer undergo interference.

Several theoretical attempts have been made to explain coherence in inelastic electron scattering. Schattschneider and co-workers [15,16] attributed the limited coherence of plasmon loss electrons to the different plasmon wavevectors that can be excited. Although difficult to measure in practice, this implies that a single plasmon event would be intrinsically coherent. Van Dyck *et al.* [17] concluded that an electron hologram is a near-perfect energy filter (∼10^−15 ^ eV energy resolution), since an energy offset ΔE between reference and object waves causes beating at a frequency ΔE/h, where h is Planck’s constant. Any interference effects are therefore destroyed over a typical measurement time (e.g. 1 s). However, coherence is preserved when both arms of the interferometer pass through the sample, and the same energy loss event occurs for both waves. While this has been experimentally validated for plasmons [9–11], it does not explain the loss of coherence with increasing energy loss. Kisielowski *et al.* [18] proposed the phase shift between propagating elastic and inelastic Hüygen wavelets as a source of coherence loss. Although this model correctly predicts the expected trend between coherence and energy loss, the fact that it involves the superposition of two waves of different energies is at odds with the arguments of Van Dyck *et al.* [17].

With current models being either incomplete or contradicting one another, it is instructive to look for insight from the wave theory of light [19], which is well established. In light optics, a monochromatic point source emits a wave train over a coherence time $\tau_{c}$. At longer time scales, internal atomic collisions within the source randomize the phase of the emitted radiation. From geometric optics, provided the path difference between two rays is smaller than $l_{c}=c\tau_{c}$, where c is the speed of light, there will be a well-defined phase relationship, resulting in interference even for long measurement times. The Fourier transform of a wave train of finite duration $\tau_{c}$ is a broadband spectrum [19], such that smaller characteristic times have a broader emission line width. Applying these ideas to electron coherence, it follows that the inelastic scattering event acts as the emission source. The quantum mechanical decoherence time, where interactions with the specimen result in a mixed state that does not produce any interference [20], could potentially be equivalent to $\tau_{c}$ in light optics. For energy-filtered studies, the interactions of interest are the elastic scattering of the inelastic wave with the heavy nuclei in the solid. The decoherence time for such interactions is, however, estimated to be longer than the time taken to detect the incident electrons in a typical TEM [20]. Therefore, decoherence time on its own cannot explain the electron coherence observed in the experiment.

In this work, the inelastic collision or interaction time is proposed as the electron-optical time scale for $\tau_{c}$. The reasoning for this choice is as follows. For the high accelerating voltages in a TEM, the sample, on average, interacts with only one incident electron at a time. The ‘emission’ caused by inelastic events, therefore, occurs as discrete ‘pulses’ of duration equal to the collision time. With this definition for $\tau_{c}$, it can be shown that the electron coherence decreases monotonically with energy loss. In fact, partial coherence is an intrinsic feature of the inelastic scattering process itself and is independent of any decoherence due to subsequent interactions with the environment. Section ‘Theory of partial coherence’ discusses the mathematical theory of electron coherence, while experimental results in Section ‘Coherence volume’ present some insights into the coherence volume for inelastic scattering in the TEM.

## Methods

SrTiO_3_ single crystal substrates were purchased from PI-KEM Limited. An electron-transparent specimen in the [001] orientation was prepared using a Helios 600 focused ion-beam microscope, with the final thinning being performed at 2 kV gallium ion-beam voltage. The sample was examined at room temperature in a JEOL 2100F field emission gun TEM operating at 200 kV. A Gatan Tridiem image filter with Ultrascan 1000 CCD detector was used to acquire energy-filtered diffraction patterns at central energy losses of 0, 30, 50, 60, 80, 100, 200 and 400 eV with 20 eV energy window. The CCD exposure time was adjusted for each energy loss such that sufficient intensity was recorded without saturating the detector. The specimen thickness measured by electron energy loss spectroscopy (EELS) is 1.6 inelastic mean free paths or ∼133 nm. The slightly larger specimen thickness improves the signal-to-noise ratio of the energy-filtered diffraction patterns, particularly for high energy losses.

## Results

### Theory of partial coherence

Coherence of inelastic scattering can be analysed quantum mechanically using time-dependent perturbation theory. Unlike previous treatments, however, the perturbing potential is only of finite duration, a key requirement for partial coherence. Let $u_{i}(r_{1},\ldots ,r_{N})$ be the initial state electron wavefunction for the solid, consisting of N electrons. The wavefunctions are derived from the unperturbed, time-independent Hamiltonian for the solid [21]. For convenience, we use the set $\left\{r_{\alpha }\right\}$ to collectively denote the electron position vectors $r_{1},\ldots ,r_{N}$, so that $u_{i}(r_{1},\ldots ,r_{N})$ may be abbreviated as $u_{i}(\left\{r_{\alpha }\right\})$. The initial state energy is $\hbar \omega_{i}$, where ℏ is Planck’s reduced constant and $\omega_{i}$ is the angular frequency. A perturbation H′(t) (see below) is applied at time t=0. From first-order perturbation theory, the time evolution of the specimen wavefunction $u(\{r_{\alpha }\},t=t\prime )$ is expressed as:


(1) $$u(\{r_{\alpha }\},t')\approx u_{i}(\left\{r_{\alpha }\right\})+c(t')u_{f}(\left\{r_{\alpha }\right\})$$

where $u_{f}(\{r_{\alpha }\})$ is the final state, many-body electron wavefunction with energy $\hbar \omega_{f}$. The final state is assumed to be an eigenstate of the unperturbed Hamiltonian for the solid. The coefficient c(t′) must be sufficiently small for perturbation methods to apply. It can be shown that [22]:


(2a) $$c(t')=\frac{1}{i\hbar }\int_{0}^{t^{'}}H_{if}(t)e^{i\omega_{if}t}dt$$

(2b) $$H_{if}(t)=\int u_{f}^{*}(\left\{r_{\alpha }\right\})H^{'}(t)u_{i}(\left\{r_{\alpha }\right\})dr_{1}\ldots dr_{N}$$

with $\omega_{if}=\omega_{f}-\omega_{i}$ and the asterisk symbol in the transition matrix element $H_{if}$ denoting the complex conjugate. For our case, H′(t) is the Coulomb interaction between the incident high-energy electron and solid-state electrons over the collision time Δt:


(3) $$H^{'}(t)=(\frac{q_{e}^{2}}{4\pi \epsilon_{o}}\sum_{\alpha }\frac{1}{\left|r-r_{\alpha }\right|})\mathrm{rect}(\frac{t-\frac{\Delta t}{2}}{\Delta t}),$$

where $q_{e}$ is the electron charge, $\epsilon_{o}$ the permittivity of free space and r is the position vector of the incident electron. rect(x) is a rectangular ‘top hat’ function which has a value of unity for |*x*| < 1/2 and zero for |*x*| > 1/2. The Coulomb interaction between the incident electron and heavy nuclei in the solid does not appear in Eq. (3), since the nuclei act as momentum reservoirs and orthogonality of $u_{i}$, $u_{f}$ states result in zero contribution to the transition matrix element $H_{if}$ [23].

For calculating the far-field scattering cross-section (Eq. 11), it is convenient to consider perturbation due to the q-Fourier component of the Coulomb kernel:


(4) $$H_{q}^{'}(t)=\frac{q_{e}^{2}}{4\pi \epsilon_{o}}(\sum_{\alpha }\int \frac{e^{-iq\cdot r}}{\left|r-r_{\alpha }\right|}dr)\mathrm{rect}(\frac{t-\Delta t/2}{\Delta t}).$$

We now make use of the following inverse Fourier transforms:


(5a) $$\frac{1}{\left|r-r_{\alpha }\right|}=\frac{1}{2\pi^{2}}\int \frac{e^{iq'\cdot (r-r_{\alpha })}}{{q'}^{2}}dq'$$

(5b) $$\mathrm{rect}(\frac{t-\Delta t/2}{\Delta t})=\frac{\Delta t}{2\pi }\int_{-\infty }^{\infty }\mathrm{sinc}(\frac{\omega \Delta t}{2})e^{i\omega (t-\frac{\Delta t}{2})}d\omega =\frac{\Delta t}{\pi }\int_{0}^{\infty }\mathrm{sinc}(\frac{\omega \Delta t}{2})c\mathrm{os}[\omega (t-\frac{\Delta t}{2})]d\omega$$

The integral in Eq. (5a) is over all vectors q'. From Eqs. (2a), (2b) and (4), the transition coefficient at the end of the collision time Δt then becomes:


(6) $$c(\Delta t)\propto \;\Delta t\int_{0}^{\Delta t}\left\{\int u_{f}^{*}{(\left\{r_{\alpha }\right\})e}^{-iq\cdot r}(\sum_{\alpha }\frac{e^{iq^{'}\cdot (r-r_{\alpha })}}{{q^{'}}^{2}})\mathrm{sinc}(\frac{\omega \Delta t}{2})\times \cos[\omega (t-\frac{\Delta t}{2})]u_{i}(\left\{r_{\alpha }\right\})dq^{'}d\omega drdr_{1}\ldots dr_{N}\right\}e^{i\omega_{if}t}dt$$

Performing the integral over time gives:


(7) $$\int_{0}^{\Delta t}\cos[\omega (t-\frac{\Delta t}{2})]e^{i\omega_{if}t}dt=\frac{1}{2}\int_{0}^{\Delta t}\left[e^{i\omega (t-\frac{\Delta t}{2})}+e^{-i\omega (t-\frac{\Delta t}{2})}\right]e^{i\omega_{if}t}dt=\frac{1}{2}[\frac{e^{-i\omega \frac{\Delta t}{2}}(e^{i(\omega +\omega_{if})\Delta t}-1)}{\omega +\omega_{if}}+\frac{e^{i\omega \frac{\Delta t}{2}}(e^{i(\omega_{if}-\omega )\Delta t}-1)}{\omega_{if}-\omega }]$$

The first term within the square brackets represents stimulated emission, while the second term is absorption [22]. We ignore the former, since we are interested in frequencies in the region $\omega \approx \omega_{if}$, where the absorption term dominates. Next, consider the integration over all vectors r in Eq. (6):


(8) $$\int {e^{-iq\cdot r}e}^{iq'\cdot (r-r_{\alpha })}dr=e^{-iq'\cdot r_{\alpha }}\delta (q'-q),$$

where δ is the Dirac delta function. Eqs. (6)–(8) lead to:

$\frac{\partial c(\Delta t)}{\partial q\partial \omega }\propto \Delta t\mathrm{sinc}(\frac{\omega \Delta t}{2})\frac{S(q)}{q^{2}}[e^{i\omega \frac{\Delta t}{2}}\frac{\left(e^{i(\omega_{if}-\omega )\Delta t}-1\right)}{\omega_{if}-\omega }]$ (9a)


(9b) $$S(q)=\sum_{\alpha }\int u_{f}^{*}(\{r_{\alpha }\})e^{-iq\cdot r_{\alpha }}u_{i}(\{r_{\alpha }\})dr_{1}\ldots dr_{N}$$

S(q) is the dynamic form factor for inelastic scattering [24]. The double-differential scattering cross-section is given by:


(10) $$\frac{\partial \sigma }{\partial q\partial \omega }\propto {\left|\frac{\partial c(\Delta t)}{\partial q\partial \omega }\right|}^{2}$$

We finally obtain:


(11) $$\frac{\partial \sigma }{\partial q\partial \omega }\propto {\left(\Delta t\right)}^{2}{\mathrm{sinc}}^{2}(\frac{\omega \Delta t}{2})\frac{{\left|S(q)\right|}^{2}}{q^{4}}[\frac{\sin^{2}[(\omega_{if}-\omega )\frac{\Delta t}{2}]}{{\left(\omega_{if}-\omega \right)}^{2}}]$$

Equation (11) contains much of the physics of inelastic scattering. The ${\left|S(q)\right|}^{2}/q^{4}$ term also appears in previous theories of inelastic scattering where the scattering time is infinitely long [24]. The dynamic form factor determines any selection rules for inelastic scattering, such as the dipole selection rule for small q. Next, consider the collision time. Due to the ${\mathrm{sinc}}^{2}(\frac{\omega \Delta t}{2})$ term, only frequencies up to ω∼2π/Δt correspond to any appreciable inelastic scattering. Therefore, the average energy loss increases for short collision times, consistent with the energy–time form of the uncertainty principle. As an example, the collision time for a 20eV energy-loss plasmon event is ∼0.2 femtoseconds. The square bracket term in Eq. (11) determines the partial coherence. The frequency spread $\Delta \omega =\omega_{if}-\omega$ is given by Δω∼2π/Δt. It is worth noting that the spread in frequencies does not contradict the energy conservation law. Rather, it is simply the result of a finite collision time and the necessary broadening that must follow when expressed in the Fourier domain, similar to the previous discussion on $\tau_{c}$ and emission line width [19]. For very long Δt, the square bracket term approximates to a delta function, and $\omega =\omega_{if}$ is strictly satisfied. Note that the spread of frequencies and temporal profile of the inelastic collision are related by a Fourier transform, so that similar conclusions on partial coherence are valid when the time-dependence of the interaction is different to the ‘rect’ function used in Eq. (3).

It is clear the inelastic collision time is similar to the coherence time $\tau_{c}$ for an optical light source. The coherence length ($l_{c}$) for inelastic scattering is therefore $l_{c}∼v\Delta t$, where v is the speed of the incident electron (it is assumed the energy loss is sufficiently small to produce negligible change in v). Note that $l_{c}$ is the temporal coherence length; spatial coherence lengths are discussed in Section ‘Coherence volume’. Since ω∼2π/Δt, we have:


(12) $$l_{c}∼\lambda /\theta_{E}$$

where λ is the electron wavelength, and the characteristic scattering angle $\theta_{E}$ for energy loss ΔE is $\theta_{E}=\Delta E/2E_{o}$. In Eq. (12), we have used the non-relativistic expression $E_{o}=mv^{2}/2$ for the incident electron energy (m is the electron mass), and the de Broglie wavelength λ is not corrected for the mean inner potential of the solid. See Supplementary Material for a derivation. Diffraction from a set of (hkl) planes with inter-planar spacing $d_{\mathrm{hkl}}$ requires that $l_{c}>d_{\mathrm{hkl}}$, or equivalently $\theta_{E}<{2\theta }_{B}$, i.e. the characteristic scattering angle must be smaller than twice the Bragg angle $\theta_{B}$. Similar qualitative arguments have been put forward by Egerton [14]. The inverse relationship between coherence length and energy loss (Eq. (12)) is consistent with previous experimental measurements [12,13].

Before concluding this section, we make one final observation. Equation (4) indicates that all electron beam positions r can potentially contribute to the perturbing potential $H_{q}^{\prime }(t)$. In practice, however, due to the oscillating exp(-iq⋅r) phase term, non-dipole electronic transitions, which have peak intensity at large q are spatially localized, while q→0 dipole transitions are delocalized [25]. The inelastic event is, therefore, effectively an extended source, especially at small q. Apart from temporal coherence, which is represented by the square bracket term in Eq. (11), we must also consider the effect of source size. Consider Fig. 1, which shows inelastic scattering from two points *A* and *B* along the far-field scattering vector q (scattering angle α). If *A* and *B* are independent point sources, for interference, the path difference *AC* must be smaller than the temporal coherence length, i.e. $r\alpha =r(\lambda q)<l_{c}$ or equivalently ${\left(qr\right)}_{\max}∼l_{c}/\lambda$. This sets an upper limit to the source size. However, Eq. (11) indicates the temporal coherence to be independent of source size. This contradiction is because we have incorrectly assumed that scattering centres *A* and *B* are independent. In fact, they are correlated, since in both cases the initial and final state wavefunctions $u_{i}$ and $u_{f}$ are identical. Therefore, coherence in inelastic scattering is determined entirely by the collision time and does not depend on delocalization.

**Fig. 1.**
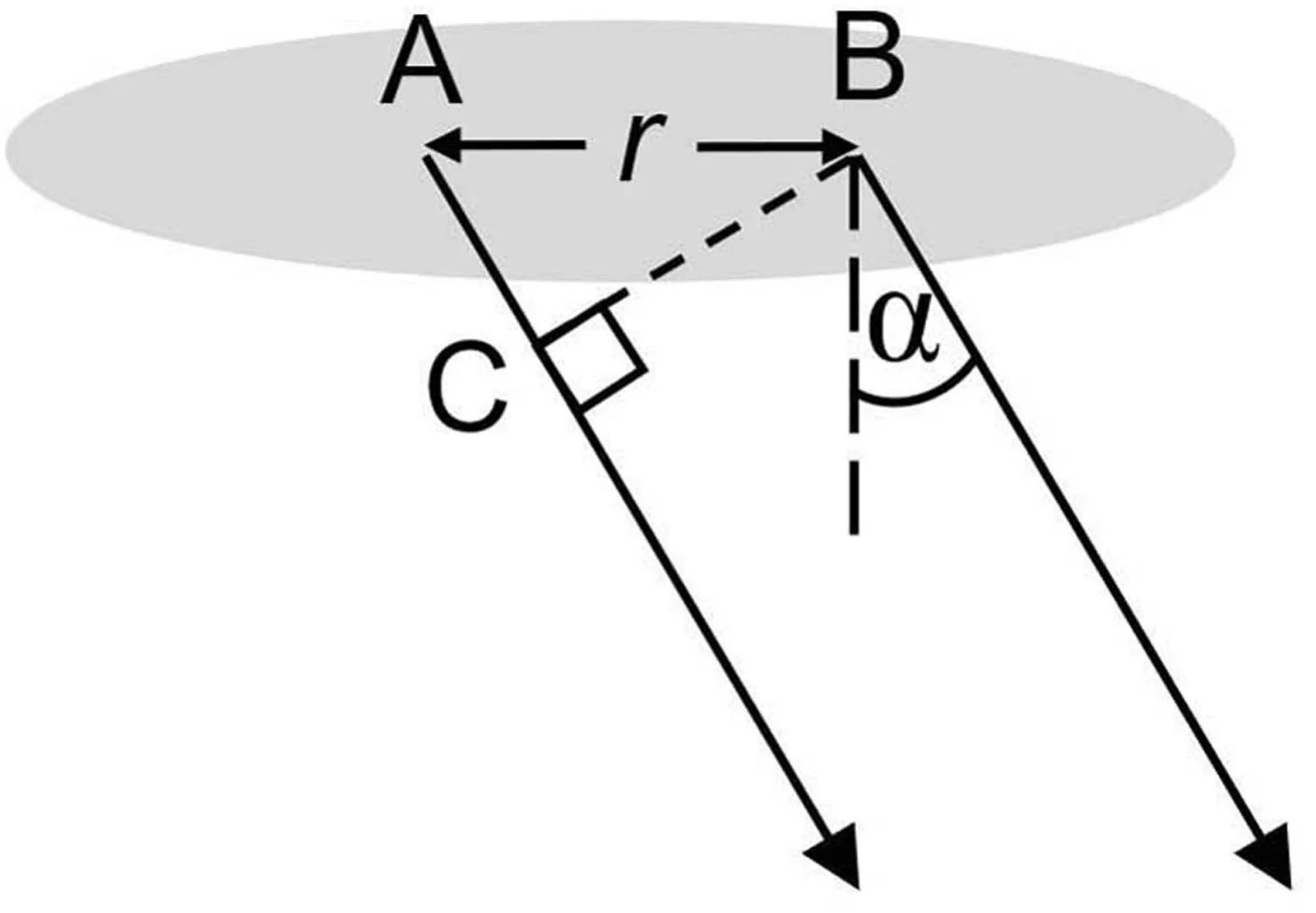
Schematic of a delocalized inelastic scattering event, represented by the shaded area. *A* and *B* are two source points that emit an inelastic wave into the far-field at the scattering angle α. The distance *AC* is the path difference between the two rays.

### Coherence volume

Experimental results on energy-filtered diffraction in SrTiO_3_ provide insight into the coherence volume for inelastic scattering. The valence EELS spectrum for SrTiO_3_ (Fig. 2) is dominated by plasmons and inter-band transitions [26], which enables the collection of energy-filtered diffraction patterns with appreciable intensity across a wide range of energy losses. [001] zone-axis diffraction patterns centred at 0, 30, 50, 100, 200 and 400 eV energy loss are shown in Fig. 3 (the full set of results can be found in the Supplementary Material). The coherence lengths $l_{c}$ for the different energy losses, as calculated by Eq. (12), are 33.4 nm (30 eV), 20.1 nm (50 eV), 10.0 nm (100 eV), 5.0 nm (200 eV) and 2.5 nm (400 eV). Note that the ∼133 nm specimen thickness (Section ‘Methods’) is such that there will be some multiple inelastic scattering within the energy-filtered window, so that the above $l_{c}$ values, which are based entirely on single inelastic scattering, are a lower limit. With increasing energy loss, the Bragg ‘spots’ become broader due to an increase in the inelastic scattering angle [27]. The larger background results in a lower contrast for the Bragg reflections. At 200 eV energy loss, a Bethe ridge (circled in Fig. 3e) appears [14], shifting to higher momenta at 400 eV (Fig. 3f), consistent with the kinematics of Compton scattering [28]. At these higher energy losses, the weaker 100 and 010 Bragg reflections are also not discernible.

**Fig. 2.**
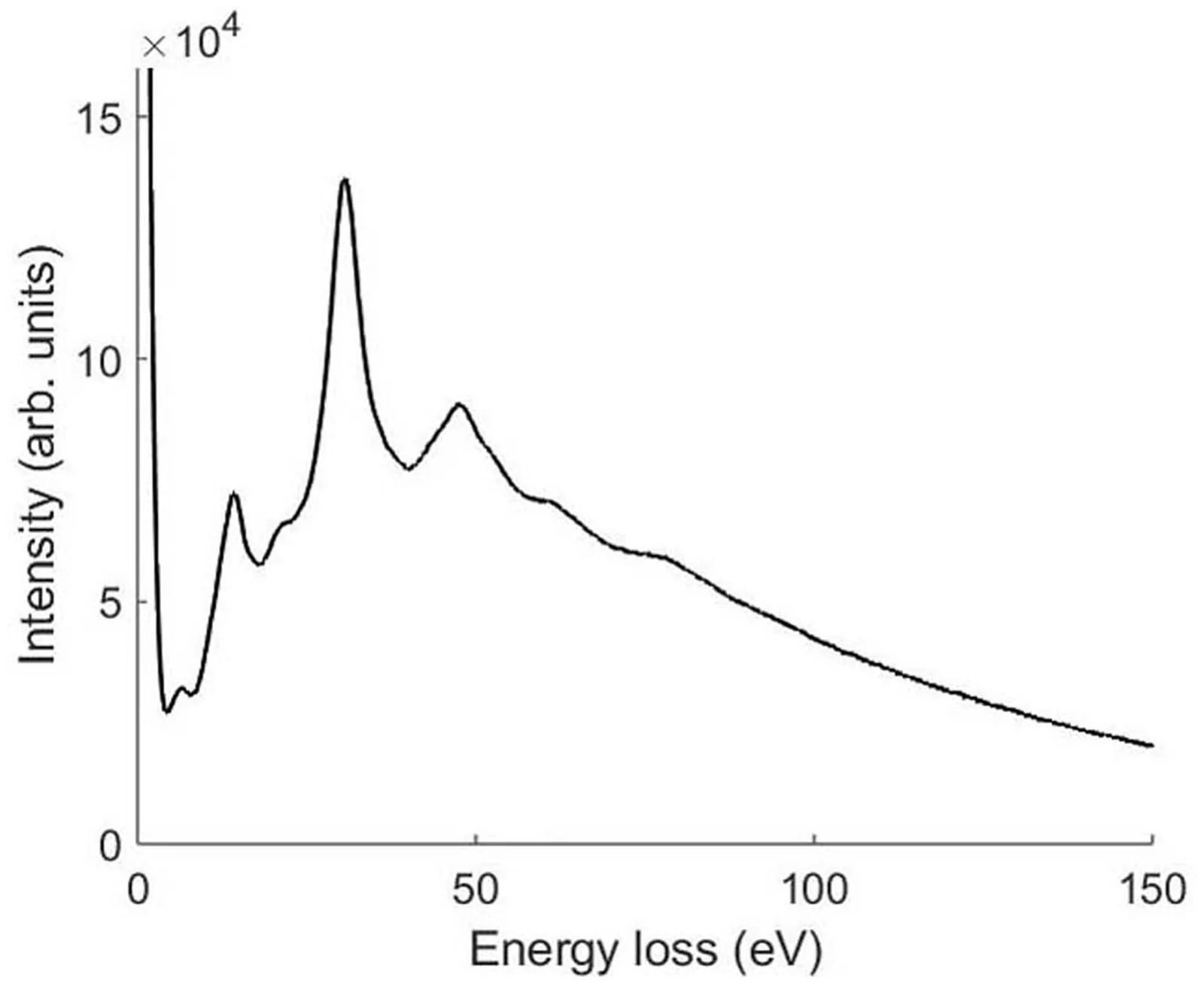
Valence EELS spectrum for the SrTiO_3_ sample. The zero-loss peak has been truncated to improve visibility of the low-energy loss region.

**Fig. 3.**
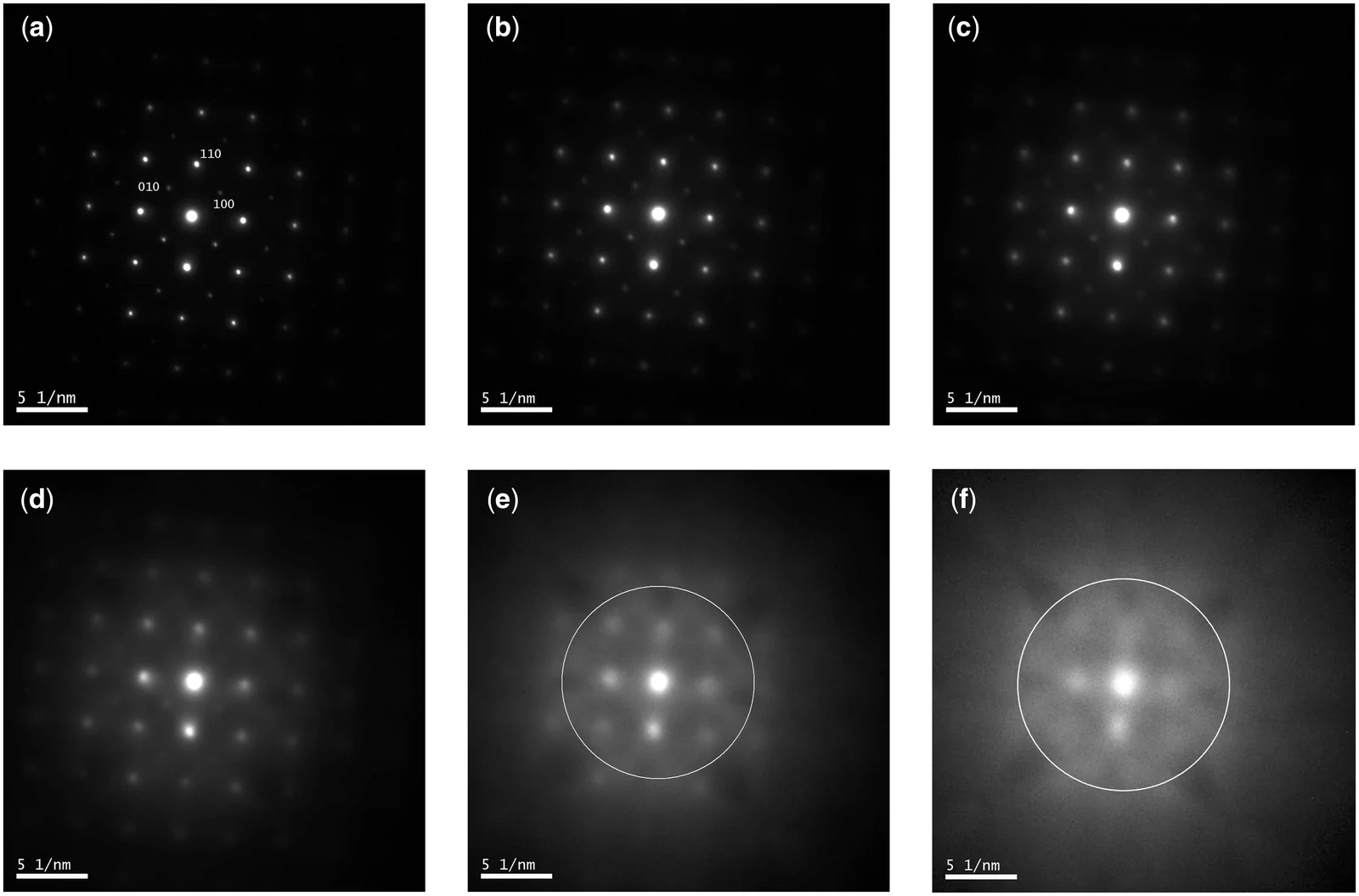
Energy-filtered diffraction patterns of [001]-SrTiO_3_ centred at (a) 0 eV, (b) 30 eV, (c) 50 eV, (d) 100 eV, (e) 200 eV and (f) 400 eV energy loss. A 20 eV energy window was used to acquire the diffraction patterns. The circles in (e) and (f) identify the Bethe ridge due to Compton scattering.

Visual inspection of Fig. 3 reveals that a significant amount of diffraction information is still preserved even at 100 eV energy loss, although the (single inelastic scattering) coherence length $l_{c}$ has decreased to ∼10% of the specimen thickness. In Fig. 4, the intensity profiles along the *h*00 and *hh*0 systematic rows are superimposed for energy losses up to 100 eV (200 and 400 eV data are omitted, since the profiles are altered by the Bethe ridge). The peak intensity of the 000 unscattered beam is normalized for direct comparison of different energy losses. The normalized intensity for strong Bragg reflections, such as 110 and 200, is approximately independent of energy loss, taking measurement error into account. The weaker 100 Bragg reflections are severely distorted, since they are superimposed on the long tails of neighbouring strong Bragg beams, especially at higher energy losses. This is consistent with previous observations of energy filtering having a large effect on Bragg reflections with a small structure factor [29]. However, after removing the background, the normalized intensity of the weak reflections is also found to be approximately independent of energy loss. A full statistical analysis at different specimen thicknesses can be found in the Supplementary Material. Considering the large decrease in coherence length $l_{c}$ over a 100 eV energy loss range, it is surprising that such a high level of quantitative agreement should still be preserved in the diffraction data. There is significant dynamical diffraction along the [001] SrTiO_3_ zone-axis, so that any reasonable change in the coherence length should have a marked effect. It has been proposed that inelastic scattering and the presence of solvent suppress dynamical diffraction in protein crystals [30]. The former, however, assumed complete loss of coherency upon inelastic scattering and treated the incident electrons as particles. This simplified model is not consistent with the quantum mechanical treatment presented here nor the experimental results for SrTiO_3_.

**Fig. 4.**
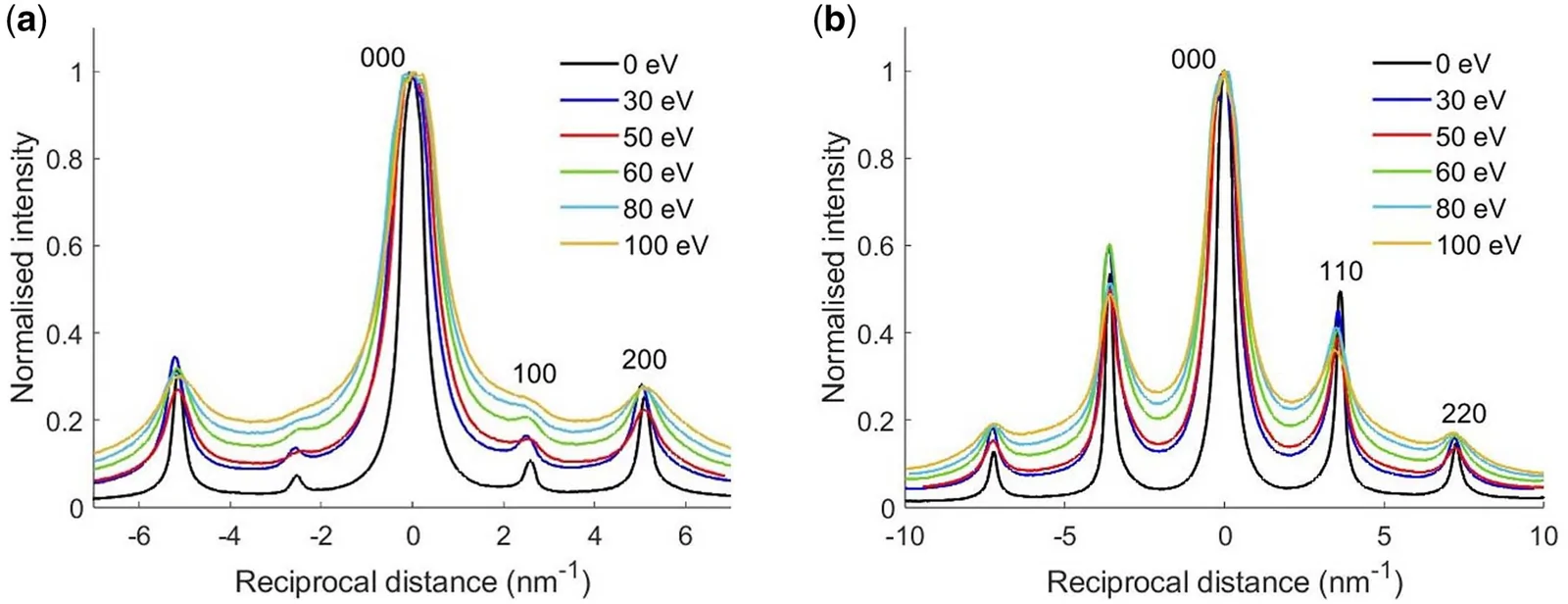
Intensity profiles for the (a) *h*00 and (b) *hh*0 systematic rows in [001]-SrTiO_3_. The line profiles were extracted from the energy-filtered diffraction patterns in Fig. 3. The 000 unscattered beam peak intensity has been normalized for direct visual comparison.

To understand the above results, we need to examine the coherence volume for inelastic scattering more closely. In particular, the shape of the coherence volume parallel and perpendicular to the incident electron beam direction is important. Consider the schematic in Fig. 5a, which shows an inelastic Hüygen wavelet generated at point *P*. The atoms in the crystal act as individual slits in a three-dimensional diffraction grating. For interference, the inelastic wave from *P* must coherently illuminate multiple slits or atoms. Two such rays, *PQ* and *PR*, that illuminate the first row of atoms are shown in Fig. 5a. Since the angle α between *PQ* and *PR* is small for inelastic scattering [14], the path difference (*PR-PQ*) is approximately equal to *QR*. It then follows that the transverse coherence volume dimension ($d_{t}$) is approximately the temporal coherence length, i.e.:


(13) $$d_{t}∼l_{c}.$$

**Fig. 5.**
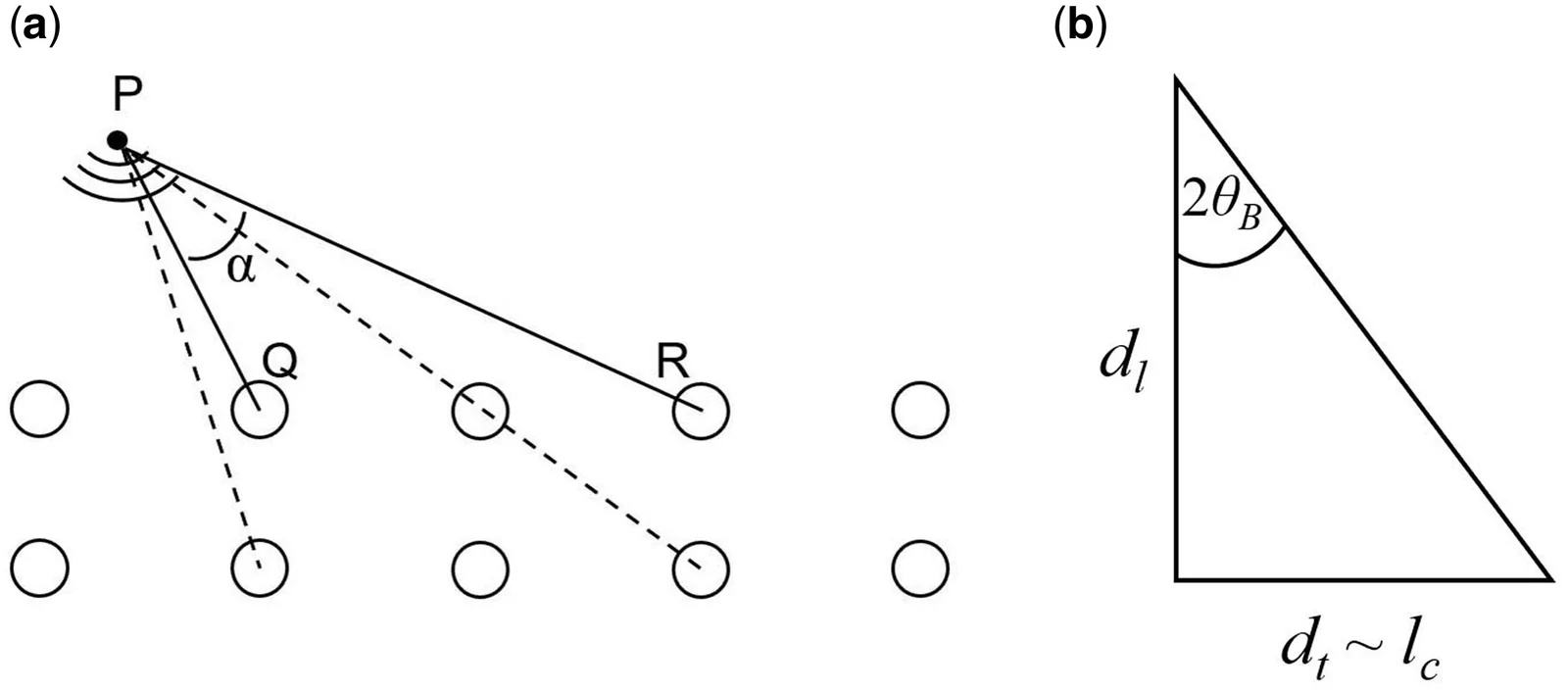
(a) Schematic for analysing coherence volume. An inelastic Hüygen wavelet is emitted from the point ‘P’. The open circles represent individual atoms in the crystal. See text for further details. (b) Geometric relationship between coherence volume dimensions along transverse ($d_{t}$) and longitudinal ($d_{l}$) directions. $\theta_{B}$ is the Bragg angle.

If $d_{t}$ or $l_{c}$ is larger than the inter-planar spacing of the diffracting planes, multiple slits (atoms) in the diffraction grating (crystal) are coherently illuminated, resulting in coherent Bragg scattering. Next, consider the second row of atoms in Fig. 5. To maintain the same transverse coherence, the angular separation between the two illuminating rays must be made smaller (dashed lines in Fig. 5a). In principle, the angular separation can be made arbitrarily small for a Hüygen wavelet, and, therefore, it would appear that there is no limit to the coherence volume dimension $d_{l}$ along the longitudinal direction. However, due to the periodic nature of the crystal, over large distances, only rays with angular separation ${2\theta }_{B}$ are sustained, so that $d_{l}$ is, in fact, of finite length. The geometric relationship between $d_{l}$ and $d_{t}$ is schematically illustrated in Fig. 5b. It follows that:


(14) $$d_{l}∼\frac{l_{c}}{{2\theta }_{B}}.$$

Since the Bragg angle $\theta_{B}$ is small, $d_{l}\gg d_{t}$. As an example, for the 200 reflection in SrTiO_3_ at 100 eV energy loss, $d_{l}$ ∼ 780 nm. This is significantly larger than $d_{t}$ ∼ 10 nm and even the specimen thickness (133 nm). It explains why the line profiles in Fig. 4 show such good quantitative agreement, despite a coherence length $l_{c}$ smaller than the specimen thickness. The absence of weak 100 Bragg reflections in the 200 eV energy loss diffraction pattern (Fig. 3e) is nevertheless unexpected. For this example, $d_{t}$ is ∼5 nm, larger than the (100) inter-planar spacing, and $d_{l}$ is ∼780 nm, much larger than the specimen thickness. Artefacts are likely to be present from other factors, such as a 20 eV energy window for acquiring the energy-filtered diffraction patterns as well as the presence of dislocations in the sample (see Supplementary Material). Together with the intrinsically weak intensity of 100 Bragg scattering, the signal-to-noise ratio during acquisition may not have been sufficiently high to observe these reflections.

The coherence volume for inelastic scattering is highly elongated along the longitudinal direction so that for most specimen thicknesses, only the transverse coherence is important. This is similar to the coherence volume in elastic scattering. Diffraction contrast analysis of defects relies on the so-called ‘column approximation’, where the diffracting volume is approximated as a narrow column extending all the way through the specimen thickness [31]. Even for a high-angle annular dark-field detector, where transverse coherence is efficiently suppressed, the longitudinal coherence is still infinitely long for elastic scattering [2]. With inelastic scattering, the longitudinal coherence (Eq. (13)) becomes finite but is nevertheless still extremely large. Once an incident electron undergoes inelastic scattering, the inelastic wave can, therefore, continue to coherently diffract as it propagates through the rest of the crystal. For small energy losses, the dynamical scattering of the inelastic wave is similar to its elastic counterpart [8,32]. In particular, only intra-band Bloch transitions are allowed [8]. This means that the electron beam intensity following a low-energy loss inelastic scattering event is approximately a continuation of the elastic pendellösung, as demonstrated in Fig. 4.

The implications of inelastic partial coherence in experiments will now be discussed. First, consider energy-filtered TEM or EELS spectrum imaging of the chemical element distribution within the specimen. The elongated coherence volume means that diffraction both prior to and after the energy loss event can influence the inelastic wavefunction exiting the specimen. Contrast reversals of the lattice structure are therefore possible [12], and a full quantification requires double channelling simulations [33]. Moving onto diffraction-based techniques, it has been observed that energy filtering can improve crystal structure refinement in 3D electron diffraction measurements [34,35], while there are also reports of no observable change [36]. Although the results for SrTiO_3_ agree with the former, particularly at low energy losses, it should be noted that ­incorrect subtraction of the diffuse background can lead to errors in the diffracted beam intensity [27]. Energy filtering largely eliminates the background intensity, thereby potentially improving quantification. Diffuse scattering due to plasmons and other low-energy loss events with large scattering cross-sections alters the distribution of transmitted electrons at small scattering angles [37,38], which may impact bright field and annular bright field imaging modes in 4D scanning TEM. Finally, plasmon excitations have a negligible effect in ptychographic phase reconstructions of gold nanoparticles [39] and graphite flakes [40], suggesting coherence properties similar to the elastic channel.

## Conclusions

Our investigation into partial coherence in inelastic scattering has uncovered some fundamental physics about the phenomenon. The quantum mechanical origin of partial coherence is explained using time-dependent perturbation theory, where, unlike previous treatments, the collision is of finite duration. The inelastic collision time emerges as the key property that governs the coherence length. Higher energy transfers have shorter collision times and therefore reduced coherence lengths. An inverse relationship between temporal coherence length and energy loss is theoretically predicted, consistent with previous measurements in the literature. Furthermore, there is no connection between coherence and inelastic scattering impact parameter, i.e. delocalization does not give rise to an extended source of incoherent ‘emitters’.

Energy-filtered diffraction measurements on [001]-oriented SrTiO_3_ show that the relative intensities of Bragg reflections remain unchanged to within measurement error up to energy losses of ∼100 eV, although the temporal coherence length is only a fraction of the specimen thickness. This is due to the anisotropic shape of the coherence volume for inelastic scattering. It is shown that the coherence along the transverse direction is given by the temporal coherence length, while the longitudinal coherence is significantly broadened (by the inverse of the Bragg angle). For most experimental conditions, the longitudinal coherence is larger than the specimen thickness, so that transverse coherence is the limiting parameter.

## Supplementary Material

dfag020_Supplementary_Data
