# Supplementary material for: Partial coherence of inelastically scattered electrons in transmission electron microscopy
Source: Microscopy (Oxf). 2026 Mar 25;75(5):390–7. doi: 10.1093/jmicro/dfag020 (PMC13652546; doi:10.1093/jmicro/dfag020)
Supplement: Supplementary file 1 [file dfag020_supplementary_data.docx]

**Supplementary Material: Partial coherence of inelastically scattered electrons in transmission electron microscopy**

BG Mendis

**(1) Derivation of coherence length *l_c_***

From $l_{c}\sim v\Delta t$ and $\omega\sim2\pi/\Delta t$ we have:

$$l_{c}\sim\frac{2\pi v}{\omega}$$

… (S1)

Substituting $\theta_{E}=\Delta E/2E_{o}=\hbar\omega/2E_{o}$ in the above equation gives:

$$l_{c}\sim\left( \frac{hv}{2E_{o}} \right)\frac{1}{\theta_{E}}$$

… (S2)

Simplifying using $E_{o}=mv^{2}/2$ and $\lambda=h/mv$ leads to the desired result:

$$l_{c}\sim\frac{\lambda}{\theta_{E}}$$

… (S3)

**(2) Energy filtered diffraction patterns**

The complete set of energy filtered diffraction patterns acquired from [001]-SrTiO_3_ are shown below.

|  |  |
| --- | --- |
| (a) | (b) |
|  |  |
| (c) | (d) |
|  |  |
| (e) | (f) |
|  |  |
| (g) | (h) |

**Figure S1:** Energy filtered diffraction patterns of [001]-SrTiO_3_ centred at (a) 0 eV, (b) 30 eV, (c) 50 eV, (d) 60 eV, (e) 80 eV, (f) 100 eV, (g) 200 eV and (h) 400 eV energy loss. A 20 eV energy window was used to acquire the diffraction patterns.

**(3) Relative intensity of Bragg reflections as a function of energy loss**

To extract relative intensities from a line profile such as Figure 4 (main text) a single Voigt function was fitted to each of the Bragg peaks. A Voigt function was chosen because the intensity of inelastic scattering typically follows a Lorentz distribution. The Gaussian part of the Voigt function represents the point spread function of the detector. The 000 unscattered beam profile was used to estimate optimum Voigt function parameters. Example least squares fits are shown in Figure S2. Although a Voigt function provided a satisfactory fit to the main peak of a Bragg reflection, it was less successful at reproducing the intensity tails. Hence only the peak intensities of the Bragg reflections were least squares fitted, rather than the full intensity profile.

|  |  |
| --- | --- |
| (a) | (b) |
|  |  |
| (c) | (d) |

**Figure S2:** Raw data intensity profiles (black line) and their least squares fit (red line). The 000 unscattered beam peak intensity has been normalised in the raw data. The profiles correspond to the *h*00 systematic row at (a) 0 eV and (b) 100 eV energy loss, as well as the *hh*0 systematic row at (c) 0 eV and (d) 100 eV energy loss.

Bragg beam intensities extracted from least squares fitting are free from background artefacts, at least to within the fitting accuracy. We analysed the relative intensities of 100, 200, 110 and 220 type Bragg reflections with respect to the 000 beam intensity. The average and standard deviation for each type was calculated from the four crystallographically equivalent reflections appearing along the [001] zone-axis. Figure S3 plots the average relative intensity for each reflection type as a function of energy loss; numerical values are provided in Table S1. Any changes in the relative intensity with energy loss are within measurement error, even for the weak 100 type reflections.

|  |  |
| --- | --- |
| (a) | (b) |
|  |  |
| (c) | (d) |

**Figure S3:** Average relative intensities of (a) 100, (b) 200, (c) 110 and (d) 220 type Bragg reflections as a function of energy loss. The Bragg beam intensity is expressed as a fraction of the 000 unscattered beam intensity.

**Table S1:** Average and standard deviation values for the relative intensity of Bragg reflections at different energy losses.

| Energy loss | Bragg reflection | | | |
| --- | --- | --- | --- | --- |
|  | 100 | 200 | 110 | 220 |
| 0 eV | 0.09 ± 0.03 | 0.28 ± 0.03 | 0.52 ± 0.12 | 0.14 ± 0.01 |
| 30 eV | 0.12 ± 0.03 | 0.30 ± 0.03 | 0.52 ± 0.11 | 0.16 ± 0.01 |
| 50 eV | 0.11 ± 0.02 | 0.23 ± 0.02 | 0.45 ± 0.14 | 0.14 ± 0.01 |
| 60 eV | 0.13 ± 0.03 | 0.28 ± 0.02 | 0.48 ± 0.14 | 0.16 ± 0.01 |
| 80 eV | 0.12 ± 0.03 | 0.26 ± 0.02 | 0.46 ± 0.13 | 0.17 ± 0.01 |
| 100 eV | 0.14 ± 0.03 | 0.26 ± 0.01 | 0.42 ± 0.11 | 0.17 ± 0.01 |

To further test the robustness of our conclusions, measurements were also carried out on the same sample at an intermediate stage of focussed ion-beam thinning. The intermediate sample thickness was measured by EELS to be 2.9 inelastic mean free paths or equivalently 241 nm, considerably larger than the final thickness of 133 nm (1.6 inelastic mean free paths). The elastic and ‘low loss’ energy filtered diffraction patterns are shown in Figure S4. The former was acquired using a 10 eV energy slit width, while the energy window for the latter covered the 50-110 eV energy loss range.

|  |  |
| --- | --- |
| (a) | (b) |

**Figure S4:** Energy filtered diffraction patterns for [001]-SrTiO_3_ intermediate thickness specimen. (a) is the elastic signal (10 eV slit width) and (b) is the ‘low loss’ signal covering the 50-110 eV energy loss range.

Representative Voigt fitting profiles for the *h*00 and *hh*0 systematic rows are shown in Figure S5, while the relative Bragg beam intensities are summarised in Table S2. Only 110 reflections have appreciable intensity, while the other reflections are an order of magnitude weaker. For these weak reflections with small peak-to-background ratio, inaccurate background modelling using a simple Voigt function can cause large systematic errors in the extracted Bragg beam intensity. This is especially true for the ‘low loss’ signal, where the background intensity is comparatively large (see Figure S5b). It is therefore not surprising that the relative intensities of the weak reflections are somewhat different for the two energy filtered diffraction patterns. This is however not the case for the stronger 110 reflections where background subtraction is not a significant issue. The relative Bragg beam intensities are therefore independent of energy loss for this thicker specimen as well, at least for the strong reflections where measurements artefacts are minimal.

|  |  |
| --- | --- |
| (a) | (b) |
|  |  |
| (c) | (d) |

**Figure S5:** Raw data intensity profiles (black line) and their least squares fit (red line) for the intermediate thickness specimen. The 000 unscattered beam peak intensity has been normalised in the raw data. The profiles correspond to the *h*00 systematic row at (a) elastic and (b) ‘low loss’, as well as the *hh*0 systematic row at (c) elastic and (d) ‘low loss’.

**Table S2:** Average and standard deviation values for the relative intensity of Bragg reflections (intermediate thickness specimen) at different energy losses.

| Energy loss | Bragg reflection | | | |
| --- | --- | --- | --- | --- |
|  | 100 | 200 | 110 | 220 |
| Elastic | 0.010 ± 0.002 | 0.054 ± 0.006 | 0.143 ± 0.040 | 0.022 ± 0.001 |
| ‘Low loss’ | 0.036 ± 0.002 | 0.090 ± 0.006 | 0.178 ± 0.026 | 0.052 ± 0.004 |

**(4) Dislocations in SrTiO_3_ sample**

Figure below indicates presence of dislocations in the SrTiO_3_ sample.

**Figure S6:** Many-beam bright-field TEM image showing dislocation line defects due to diffraction contrast.
